# Some Cases in Ophthalmology

**Published:** 1887-03

**Authors:** W. L. Bullard

**Affiliations:** Specialist in Diseases of Eye, Ear, Nose and Throat, Columbus, Ga.


### SOME CASES IN OPHTHALMOLOGY.

BY DR. W. L. BULLARD,
Specialist in Diseases of Eye, Ear, Nose and Throat, Columbus, Ga.

Miss P. C——, æt twelve, student, consulted me two years ago for squint. The first notice of the eyes being cross was five years before. Since she commenced school, and using the eyes for near work, has suffered no little from headache. With retinoscopy verified with the ophthalmoscope and ordinary test glasses, I found, besides being cross-eyed, that patient had compound hypermetropic astigmatism of both eyes to this effect:

O. S. $\frac{6}{24}$ plus 2 D. O plus C. 4 D 70°. Equal $\frac{6}{6}$

O. D. $\frac{6}{24}$ plus 2 D. O plus C. 4 D 95°. Equal $\frac{6}{6}$

I advised tenotomy and proper glasses. Patient consented to the operation, but for fear of being teased at school would not consent to wear glasses. About one year after operation patient returned and wished to have her eyes refracted. Mocroscopically the eyes looked perfectly natural, yet the headache continued. The proper glasses were ordered, and in conversation with her father not long since he informed me that she would not part with her glasses under any consideration; the headache had ceased to annoy her, and she can now attend school regularly, which she could not do before she commenced to wear glasses.

Dr. R. A——, æt eighty, consulted me two years ago. On examination, I found senile cataract in both eyes; the opacity somewhat more advanced in the left, yet not fully "ripe," but it had not advanced any during the last three years, at which time patient consulted an oculist, but was refused an operation. The pupil was contracted, and its dilatation was quite limited under the influence of atropic sol., 2 grains to the drachm. Cognizant of Noyes' advice "for good prognosis, one ought to have an active pupil as well as a mature cataract," and, moreover, that it is getting out of the old rut to operate when there exist qualitative perception of light—that is, to see objects or to count fingers; I nevertheless advised an operation. Some may ask, Why such advice? In reply, I might mention that sometimes a cataract is wholly ripe and yet a patient will count fingers at one foot, with a good light. In these cases the lens is very dark, of a mahogany or deep yellow hue, very slow in developing, and should not be refused an operation. I, of course, mentioned the so-said disadvantages connected with an operation, such as inactive pupil, his age, etc.; yet I ventured—not believing in a preliminary iridectomy—to advise an extraction as the best means of relief. Patient, without much reluctance, agreed to take the chances, and promised to return in two weeks, which, however, he failed to do, but returned in about six months, and as there was no perceivable change in the lens, was fully determined to be operated upon. After cocainizing the eye, in the presence of Drs. Hunt and Bruce, of this city, I performed Græfe's modified linear extraction. The operation was quite normal—that is to say, it was not attended by any mishaps. The interesting features, especially to those who may be interested in ocular surgery, and the motive that prompt a report of the case, is in the after-treatment.

There is a diversity of opinions as to the dressing of the eye after cataract operations, iridectomies, etc. The majority of oculists, however, are still using pads and bandages. Recently, a few (Michel, Chisolm) have excluded dark rooms and reduced the dressing to a simple strip of isinglass plaster, while others, like Pagenstecher (Wiesbaden), discard all dressing—advocating the open treatment. The eye was bathed with an acid boracic solution, and satisfying myself that the iris was free, the eye was tightly padded with absorbent cotton, and a bandage of Swiss gauze, wet, and applied as advocated by Hotz. The room was darkened, but as there was no perceivable difference to the patient, it was thought unnecessary, and sufficient light was let in to make the attendants comfortable—that is to say, they could see how to move around the room, read, etc., with perfect ease. On the second day patient was allowed to sit up and walk about the room; third day, without my consent, he walked out into the back yard; fifth day entertained company, he, with the rest, sitting out in the front porch. After the fifth day bandage was left off, and shade used, instead, for a number of days. Glasses were fitted, and, as the doctor expressed it, he "could read the Atlanta Constitution, and it required good sight to do that." Distant vision with plus glass is not perfect, but with plus 20 D. J. No. 6 is read quite readily.

Mr. A. G——, æt sixty-nine, had cataract fully formed in right eye and nearly so in left. Patient healthy, and had good perception of light. Under the anæsthetic influence of a 1-per cent. sol. of cocaine mur. the right lens was removed by Græfe's linear section. Patient counted fingers readily after lens extraction. The eye was washed with a boracic acid solution, and the lids of each eye were kept closed by a light absorbent cotton pad and Swiss gauze bandage. As there seemed to be no necessity for removing the bandage it was not disturbed until the third day, when the eye was examined and a solution of atropia dropped into the eye; this was repeated for four days, when the bandage was left off, and the patient left without protection. The eye operated upon was slightly injected, but patient could look out into the street without any inconvenience. Glasses were ordered, and patient returned to his home in Alabama quite elated over his good fortune of restored sight. Nothing was said to the attendants in regard to darkening the room, and it was kept in the usual way. Patient did not complain of the light when dressing the eye.

A. S. B——, æt forty-one; blind in right eye from a lick in eye, inflicted thirty years ago. Condition of eye at the time of examination as follows: Leiucomatous spot on cornea, nasal side; iris adhered to opacity (anterior synechia); pupil occluded, from concurrent iritis; greater part of iris adhered to the lens (posterior synechia). Could get no reflex of fundus with ophthalmoscope, but a slight wavy motion by the iris indicated that the lens had been partly or wholly absorbed. Considering the patient's age, the occluded pupil—which prevented any communication between the anterior and so-called posterior chambers—the liability of further attacks of iritis—possibly lead to cyclitis or choroiditis—and the probability of the eye becoming glaucomatous, I advised an operation (iridectomy), not so much from visual motive, but as a protection to the eye from cyclitis and glaucoma. "This operation is called for in such cases because the vision of the eye is almost entirely lost, and because the communication between the posterior chamber is abolished. The secretion going on in the posterior chamber, and increasing the tension of this part, will cause pressure on the iris and result in atrophy of this body, which, at the same time, will yield to the *vis-a-tergo*, and will bulge out into the anterior chamber (bombs iris)."* After telling patient that quite likely he would suffer more or less from iritis, the eye was cocainized with a solution of cocaine mur., and an iridectomy was made upward, the adhesions torn up as far as circumstances would ad-

* Mittendorf—Diseases of the Eye and Ear, p. 172.

mit, and the lids of each eye closed and held in position by pieces of diaphanous isinglass silk plaster, as advised by Chisolm and Michel. Patient was put in a hack and sent home, five blocks away. The adhesive strip was removed on the third day. As anticipated, some iritic inflammation came on, but yielded quite readily under the influence of atropia sol. 2-per cent. The room was moderately darkened, though patient never complained of the light. Directly after the operation vision was not improved, but at this writing vision is 3.40 without a glass. The lens was found to be mostly absorbed, and since operation the absorbtion slowly continues, from above, downwards. Considerable reflex in upper part of iridectomy, but in pupillary space some opacity still exist. The patient, having one good eye, and as his vision in operated eye is improving, with a communication now existing between the anterior and posterior chambers, I am in no hurry to practice Keratonyxis.

The oculist will readily see why this case has been reported, when the occluded pupil, with posterior and anterior synechia, together with traumatic lesion of the cornea and complicated, no doubt, with ruptured lens thirty years ago, is considered.

M. K——, æt sixty-four, with senile cataract in both eyes, fully ripe. Patient healthy, with good perception of light. In the presence of Drs. Griggs and Sheridan the right eye was anæsthetized with a 1-per cent. solution of cocaine, and the lens extracted by Græfe's linear method. Patient gave no evidence of pain, and counted fingers after lens extraction. The eye was washed with boracic acid solution, light pad of absorbent cotton applied and retained by a thin strip of cloth folded and securely tied. The eye was not disturbed until the third day, when the dressing was removed and a solution of atropia, 2-per cent., instilled into the eye twice daily for four days, at which time the eye was left without any protection. The room was not darkened, and patient never complained of the light when dressing the eye, even after bandage was left off. The eye was considerably injected, yet it did not water, and in two weeks after the operation, the hyperæmia having almost disappeared, glasses were fitted and she returned to her home in the Northern part of the State, very much elated at the reality of seeing again.

The exclusion of dark rooms, not mentioning compresses and bandage, is a revolution complete in eye surgery. Friends can read for the entertainment of those operated upon, and with the anæsthetic influence of cocaine ($\frac{1}{2}$ to 1-per cent.), cataract patients need have little dread from confinement or pain.

*January 5, 1887.*

